# Distribution, Sources, and Risk Assessment of Organochlorine Pesticides in Water from Beiluo River, Loess Plateau, China

**DOI:** 10.3390/toxics11060496

**Published:** 2023-05-31

**Authors:** Jipu Guo, Wenwu Chen, Menglei Wu, Chengkai Qu, Haotian Sun, Jiahua Guo

**Affiliations:** 1 State Grid Shaanxi Electric Power Research Institute, Xi’an 710100, China; 2 Shaanxi Key Laboratory of Earth Surface System and Environmental Carrying Capacity, College of Urban and Environmental Sciences, Northwest University, Xi’an 710100, China; 3 Key Laboratory of Cultural Heritage Research and Conservation, School of Culture Heritage, Northwest University, Xi’an 710127, China; 4 State Key Laboratory of Biogeology and Environmental Geology, School of Environmental Studies, China University of Geosciences, Wuhan 430074, China

**Keywords:** organochlorine pesticides, distribution, sources, risk assessment, Beiluo River

## Abstract

The Loess Plateau has been a focus of public discussion and environmental concerns over the past three decades. In this study, in order to investigate the effect of OCP pollution in water of the Beiluo River, concentrations of 25 OCPs at 17 locations in the water were examined. The results showed that the concentration of ∑OCPs in the water ranged from 1.76 to 32.57 ng L^−1^, with an average concentration of 7.23 ng L^−1^. Compared with other basins in China and abroad, the OCP content in the Beiluo River was at a medium level. Hexachlorocyclohexane (HCH) pollution in the Beiluo River was mainly from the mixed input of lindane and technical HCHs. Dichlorodiphenyltrichloroethane (DDT) pollution was mainly from the mixed input of technical DDTs and dicofol. Most of the OCP pollution came from historical residues. The risk assessment results showed that hexachlorobenzene (HCB) and endosulfan had high ecological risks in the middle and lower reaches of the Beiluo River. Most residual OCPs were not sufficient to pose carcinogenic and non-carcinogenic health risks to humans. The results of this study can provide a reference for OCP prevention and control and watershed environmental management.

## 1. Introduction

Organic chlorine pesticides (OCPs) are typical persistent organic pollutants (POPs) with high toxicity, environmental persistence, high bioaccumulation, and low degradation rate, posing a significant threat to the environment and human health [1]. Many studies have shown that OCPs can cause severe endocrine disruption and other carcinogenic and non-carcinogenic adverse health effects, such as newborn health complications, cancer, developmental neurotoxicity, etc. [2,3]. Thus, OCPs are one of the POPs listed as a priority by the Stockholm Convention and have attracted increasing attention from researchers [4,5]. China is the second largest producer of pesticides. In the 1980s, we discovered the negative impact of OCPs on the environment and have successively banned them [6]. However, due to their persistence in the environment, OCPs have still been detected in environmental media such as water bodies, sediments, and organisms in recent years [7,8]. OCPs are diffused into the water through input routes such as non-point source runoff, wastewater discharge, atmospheric migration, and sedimentation diffusion, posing a threat to the water ecosystem [9,10]. Thus, it is of great significance to study the temporal and spatial distribution, source, and risk assessment of OCPs in the water environment for effective prevention and control of OCP pollution, protection of human health, and sustainable development of the watershed environment.

The Loess Plateau is located in the north–central region of China, which has the largest loess coverage area in the world [11]. In recent years, due to unreasonable human behavior, such as over-farming and over-grazing, soil and water loss on the Loess Plateau has become increasingly severe, and the environment is relatively fragile [12]. A number of scholars have monitored POPs on the Loess Plateau. In the process of investigating the distribution characteristics of OCPs and polychlorinated biphenyls (PCBs) in groundwater of the riparian zone of the Loess Plateau, Sun et al. found that the concentration distribution of OCPs is much higher than that of PCBs. The long-term occurrence of POPs in groundwater will cause certain ecological risks [13]. In addition, the concentration of polycyclic aromatic hydrocarbons (PAHs) in river sediments in this area ranges from 194–514 ng g^−1^, and human activities greatly influence PAH pollution [14]. However, the pollution characteristics of OCPs in river surface water in the Loess Plateau need to be better understood.

The Beiluo River is a typical river on the Loess Plateau. In recent years, the economy of this basin has developed rapidly, and new achievements have been made in agriculture, industry, and transportation. The upper reaches of the Beiluo River are rich in coal and oil resources and relatively industrially developed. Still, there are also problems, such as oil leakage and serious organic pollution [15]. The land in the middle and lower reaches is flat, the soil is fertile, and agriculture develops rapidly. As a result, agricultural products such as pesticides and fertilizers are used in large quantities, leading to serious organic pollution in the Beiluo River Basin, threatening the environment and human health in the basin [16]. Currently, studies of this basin mainly focus on monitoring indicator organisms (such as zooplankton) and water quality evaluation [16,17], while there are few studies on organic pollution [18].

Magulova et al. proposed that POP monitoring data are essential to establish a global baseline of POP levels and predict changes in POP concentrations [19]. To the best of our knowledge, previous studies have investigated PAHs and PCBs in the Beiluo River Basin, but little is known about OCP pollution in the surface water of the basin [14,18]. Therefore, monitoring and investigation of Beiluo River OCPs were necessary. The main objectives of this study are as follows: (1) to reveal the pollution levels and spatial distribution of 25 OCPs in water; (2) to analyze the potential sources of OCPs in water; and (3) to assess the ecological health risk in the study area.

## 2. Materials and Methods

### 2.1. Study Area and Sampling

**Figure 1:**
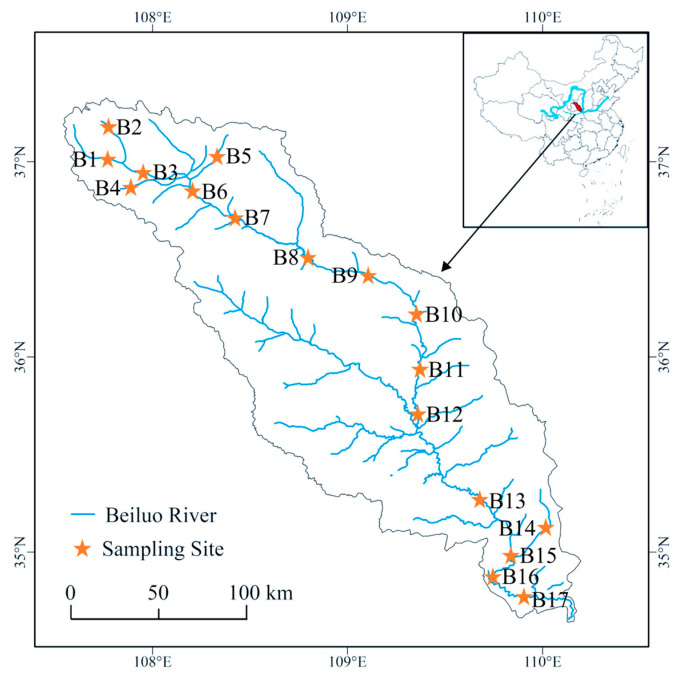
Sampling map of water in the Beiluo River.

The Beiluo River, located between 107°34′−110°01′ E and 34°57′−37°17′ N, is a first-level tributary of the Weihe River and a second-level tributary of the Yellow River, with a total length of 680 km and a drainage area of about 2.69 × 10^4^ km^2^ [14,20]. This region belongs to the temperate continental semi-arid climate belt, with average annual rainfall of 510–540 mm. The spatial distribution of precipitation is significantly different, and it is mainly concentrated in summer (June–September), and the average annual temperature is 7.5 °C [21]. This study collected water samples from the Beiluo River in June 2021. The sampling points were located on the mainstream and some tributaries of the Beiluo River, with a total of 17 sampling points, as shown in Figure 1. Water samples in the amount of 1.5 L were collected at each sampling site, put into glass bottles, brought back to the laboratory, and stored in the refrigerator at 4 °C for chemical analysis.

### 2.2. Chemical Analysis and Quality Control

The 25 OCPs targeted were identified for all samples and divided into 7 chemical subgroups: HCHs (α-HCH, β-HCH, γ-HCH, and δ-HCH), DDTs (o,p’-DDT, p,p’-DDT, o,p’-DDD, p,p’-DDD, o,p’-DDE, and p,p’-DDE), HCB, DRINs (aldrin, dieldrin, endrin, endrin aldehyde, and endrin ketone), SULPHs (endosulfan I, endosulfan II, and endosulfan sulfate), CHLs (cis-Chlordane, trans-Chlordane, heptachlor, and heptachlor epoxide isomer B), and others (methoxychlor and mirex).

The water samples were treated using the method in the Chinese national standard, “Water Quality—Determination of Organochlorine Pesticides and Chlorobenzenes—Gas Chromatography Mass Spectrometry” (HJ699-2014), and previous studies were referred to [22,23,24]. First, all the water samples were filtered, and then 1 L amounts of the water samples were added into the liquid separation funnel. The recovery indicators were 2,4,5,6-tetrachloro-m-xylene (TcmX), PCB65, and PCB155, and 25–30 mL of dichloromethane was added for extraction. The extract was concentrated to 4 mL on a rotary evaporator at 40 °C and 60 r/min. The concentrated solution was transferred to a chromatography column (alumina to silica gel ratio of 1:2, column length: 9 cm) for separation and purification. At the same time, the eluent was eluted with a 20 mL mixture (dichloromethane to n-hexane ratio of 2:3). The eluent collected was again concentrated to 0.5–1 mL using a rotary evaporator, and the concentrated solution was transferred to 2 mL cell bottles. The solution was blown to about 0.2 mL in soft, high-purity nitrogen, and 5 μL of pentachloronitrobenzene (PCNB) was added as the instrument’s internal standard. The measurements of OCPs were performed using gas chromatography–triple quadrupole mass spectrometry (GC–MS/MS) equipped with an HP-5 MS capillary column (30 m length, 0.25 mm i.d., 0.25 mm film thickness) and an electron impact (EI) ionization source.

All samples were subject to strict quality control and assurance during the analysis process. One blank sample (to test whether the sample was interfered with by instruments, reagents, and human factors), one matrix sample (to test the recovery rate of the pretreatment method), and three parallel samples (to reduce experimental errors) were required for each batch of samples. None of the components to be measured were detected in the blank sample. The surrogate recoveries were 69.46% ± 32.81%, 82.14% ± 6.46%, and 79.00% ± 12.71% for TCMX, PCB65, and PCB155, respectively. The results showed that this experiment was feasible for the treatment of samples [18].

### 2.3. Risk Assessment in the Beiluo River

#### 2.3.1. Ecological Risk Assessment

The ecological risk quotient (RQ) was used to assess the ecological risk of OCPs in the water body of the Beiluo River. The formula for calculating the RQ value is as follows [13]:

(1) $$\mathrm{RQ}=\frac{\mathrm{MEC}}{\mathrm{PNEC}}$$

(2) $$\mathrm{PNEC}=\frac{\mathrm{EC}50\;\mathrm{or}\;\mathrm{LC}50}{\mathrm{Assessment}\;\mathrm{Factor}}$$

where MEC is the measured concentration of OCPs in the water samples, and PNEC is the predicted no-effect concentration. The PNEC value is calculated by dividing the LC50 or EC50 by the Assessment Factor (1000). A RQ greater than 1 indicates a high level of ecological risk. When the RQ is 0.1–1, it indicates that the ecological risk is moderate. The ecological risk is insignificant when the RQ is less than 0.1 [25].

#### 2.3.2. Health Risk Assessment

The carcinogenic and non-carcinogenic risks of OCPs in water samples were calculated using the method recommended by the United States Environmental Protection Agency (USEPA) [26]. Different exposure routes of OCPs pose different risks to the human body, among which the main exposure routes are water intake and skin contact [27,28]. This study combined the above two exposure routes to evaluate the carcinogenic risk of OCPs in water samples. The formula for calculating risk is as follows:

(3) $$E_{1}=\frac{C\times \mathrm{IR}\times \mathrm{EF}\times \mathrm{ED}}{\mathrm{BW}\times \mathrm{AT}}$$

(4) $$E_{2}=\frac{C\times k\times \mathrm{SA}\times \mathrm{EF}\times \mathrm{FE}\times \mathrm{ED}}{500\times \mathrm{BW}\times \mathrm{AT}\times f}\sqrt{\frac{6\tau \times \mathrm{TE}}{\pi }}$$

(5) $$E=E_{1}+E_{2}$$

where E_1_ is the human body’s intake of OCPs when exposed through drinking water; E_2_ is the human body’s intake of OCPs when exposed through skin contact; E is the human body’s long-term total intake of OCPs; C is the measured concentration of OCPs in the water samples; IR is the human body’s mean daily intake of water; EF is the exposure frequency; ED is the duration of exposure; BW is the mean body weight; AT is the average time; k is the penetration parameter of human skin; SA is the surface area of the human body; FE is the bathing frequency of the human body; f is the adsorption ratio of the human intestinal tract; τ is the delay time; and TE is the contact time of human skin.

(6) R=E×SF

(7) $$\mathrm{HI}=\frac{E}{\mathrm{RfD}}$$

where R is the carcinogenic risk value; HI is the non-carcinogenic risk value; SF is the cancer coefficient; and RfD is the reference dose. The formula used to calculate the health risks refers to previous studies, and the units and values of the parameters in the formula are shown in Table S1 [5,29]. R > 1 × 10^−6^ indicates a potential carcinogenic risk, and 1 × 10^−4^ is the upper limit of acceptable cancer risk. HI > 1 indicates a potential non-carcinogenic effect [30].

### 2.4. Data Processing and Statistical Analyses

For data analysis, “not detected” OCP data are replaced with the value of 1/2 of its method detection limit (MDL) [31,32]. All statistical analyses (e.g., mean, maximum, minimum, standard deviation, and coefficient of variation) were performed using Microsoft Excel 2021. The diagnostic ratios have been widely used to investigate the origin, input history, and environmental behavior of OCPs, and they were used in this study to determine the main source of OCPs [30]. The Arcgis 10.2 program (ESRI Inc., Redlands, CA, USA) was used to draw the spatial distribution map of sampling points, while other graphs in this paper were drawn using Prism 8.0 and Origin 2021 software.

## 3. Results

### 3.1. Concentration and Distribution of OCPs in Water

**Figure 2:**
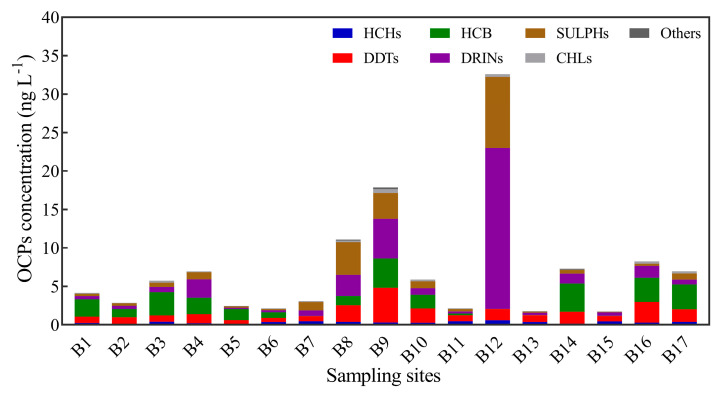
Distribution of OCPs in water of the Beiluo River.

All 25 OCPs in the water of the Beiluo River were detected to varying degrees, and Table S2 lists the concentration distribution data of OCPs in the water. OCP concentrations and distribution in the water of the Beiluo River Basin are displayed in a histogram in Figure 2. The results showed that the ∑OCP concentrations ranged from 1.76 to 32.57 ng L^−1^, and the mean concentration was 7.23 ng L^−1^, with the lowest concentration in B15 and the highest concentration in B12. Among them, the mean concentrations of DRINs are the highest, accounting for 31.94% of the ∑OCPs, while the contents of HCB and DDTs are second only to DRINs, accounting for 22.54% and 19.29%, respectively. The mean concentrations of OCPs in the middle stream of the Beiluo River (12.20 ± 11.59 ng L^−1^) were higher than those in the upstream (4.05 ± 1.94 ng L^−1^) and the downstream (5.20 ± 3.17 ng L^−1^).

Table S3 shows descriptive statistics of OCP concentrations in the Beiluo River. The coefficient of variation (CV) can reflect the spatial heterogeneity of pollutants to some extent. In general, the CV is positively correlated with the degree of interference from human activity. A CV above one is regarded as high variability [33]. The results showed that DRINs (CV = 2.15), SULPHs (CV = 1.71), methoxychlor (CV = 1.26), and mirex (CV = 2.044) displayed high variability, indicating that the OCPs above had a large dispersion and that the content was affected by human activities in the Beiluo River Basin.

### 3.2. Source Apportionment for OCPs in Water

**Figure 3:**
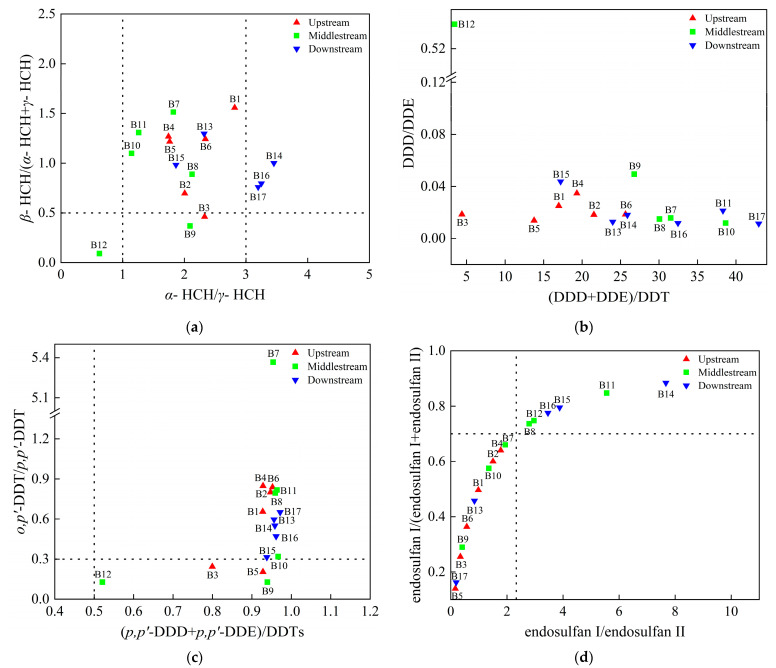
Distribution of HCHs (**a**), DDTs (**b**,**c**), and SULPHs (**d**) sources in water of the Beiluo River.

HCHs in the environment are mainly derived from the input of technical HCHs and lindane. The α-HCH/γ-HCH ratio is frequently used to identify the source of HCHs. The ratio of α-HCH/γ-HCH ranged from three to seven for technical HCHs; if the ratio of α-HCH/γ-HCH was less than or close to one, it implied the use of a lindane formulation [34]. In addition, β-HCH/(α-HCH + γ-HCH) is often used to reflect the degradation degree of HCHs so as to determine the input history of HCHs. If the ratio is less than 0.5, it means that there are newly imported HCHs in the environment. On the contrary, another study found that it means that HCHs are mainly historical pollution [35]. In this study, the α/γ-HCH ratio of B10–B12 in the middle stream was around one (Figure 3a), indicating that HCHs were mainly from lindane emission. The α/γ-HCH ratio of B14, B16, and B17 downstream was greater than three, showing that HCHs mainly came from the use of technical HCHs. However, the ratios of other samples were all in the range of one to three directly, possibly reflecting a mixed input of lindane and technical HCHs. The ratio of β-HCH/(α-HCH + γ-HCH) in most of the sampling sites was greater than 0.5, indicating that HCHs in the water were mainly derived from historical residues.

DDTs can be degraded into DDD in an anaerobic environment and DDE in an aerobic environment [36]. The DDD/DDE ratio can be used to estimate the degradation environment of DDTs [37]. The ratio of (DDD + DDE)/DDT can determine whether there are new DDT inputs [38]. As shown in Figure 3b, DDD/DDE and (DDD + DDE)/DDT ratios varied from 0.01 to 0.54 and 3.44 to 42.97, respectively. Thus, these ratios suggest that most DDTs were decomposed in an aerobic environment and mostly came from historical residues. In addition, the o,p’-DDT/p,p’-DDT ratio is frequently used to determine the source of DDTs. When the ratio is less than 0.3, DDTs come from the input of technical DDTs. When the ratio is between 0.3 and 7, DDTs are derived from technical DDTs mixed with dicofol. According to [39], the ratio of (p,p’-DDD + p,p’-DDE)/DDTs can be used to check whether there is new DDT input. If the ratio is greater than 0.5, it indicates that DDTs have been degraded completely in the environment. As shown in Figure 3c, except for B3 and B5 in the upstream and B9 and B12 in the middle stream, the o,p’-DDT/p,p’-DDT ratios at the other sampling points were between 0.3 and 7, indicating that DDTs were from technical DDTs and dicofol mainly used together. The ratio of (p,p’-DDD + p,p’-DDE)/DDTs in all samples was greater than 0.5, indicating that the degradation of DDTs was complete.

Endosulfan has been widely used as an agricultural insecticide over the past 30 years [40]. Endosulfan is a hydrophobic compound that normally adsorbs to the soil, but during heavy rains, a portion of it desorbs from the soil and enters the water environment through surface runoff [41]. Endosulfan I decomposes at a higher rate than endosulfan II in the environment, so the ratio of the two can be used to determine whether new endosulfan has been imported recently [42]. A ratio of endosulfan I/endosulfan II less than 2.3 indicates historical use of endosulfan, whereas a ratio greater than 2.3 indicates recent use [43]. In addition, when the endosulfan I/(endosulfan I + endosulfan II) ratio is close to 0.7, it indicates that new endosulfan has been imported recently [44]. The endosulfan I/endosulfan II ratio was greater than 2.3, and the endosulfan I/(endosulfan I + endosulfan II) ratio was close to 0.7 at the sampling sites, which mainly contained B7, B8, and B12 in the middle stream, indicating that endosulfan was used or imported recently in these sites, and the majority of the remaining sampling sites were historical residues.

Chlordane is an organochlorine insecticide widely used in cash crops such as sugarcane, tomatoes, potatoes, and vegetables [45]. The main constituents in technical chlordane are cis-chlordane (CC, 11%), trans-chlordane (TC, 13%), and heptachlor (HEPT, 5%) [46]. The CC/TC ratio is commonly used to determine whether chlordane was recently added. If the ratio is less than or close to one, it is considered to include recently imported chlordane; otherwise, it is historical residue. In this study, approximately 53% of the CC/TC ratio was less than one (Table S4), indicating that there was recently imported chlordane pollution in water of the Beiluo River Basin.

Heptachlors (HCB), which are typical byproducts of industrial and agricultural production, were widely produced and used in China during the 1960s and 1970s [47]. In this study, the mean concentration of HCB was 1.63 ng L^−1^ (Table S3), suggesting that the source of HCB was mainly byproducts of industrial and agricultural production. Methoxychlor and mirex mean concentrations were 0.033 ng L^−1^ and 0.0094 ng L^−1^, respectively, with low content and negligible pollution in the environment.

### 3.3. Risk Assessment for OCPs in Water

#### 3.3.1. Ecological Risk Assessment

**Figure 4:**
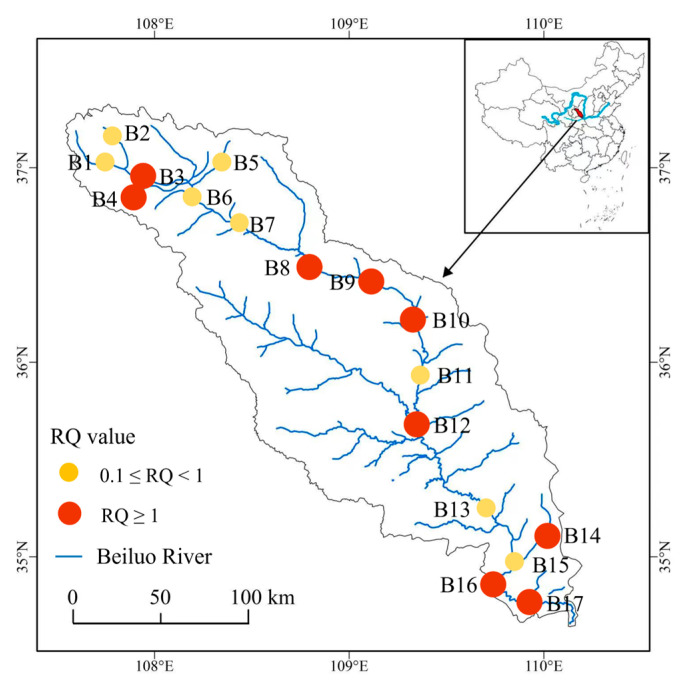
The ecological risk of OCPs in water of the Beiluo River.

OCPs can accumulate through the food chain in the adipose tissue of organisms, resulting in a biological magnification effect that poses a major hazard to top predators and has serious toxic effects on the entire ecosystem [48]. Figure 4 shows the ecological risk assessment results of OCPs in the Beiluo River using the ecological risk quotient. RQ values in the Beiluo River Basin are generally greater than 0.1, with medium ecological risks. Among them, the region with the highest ecological risks is in the middle stream of the Beiluo River. The ecological risk assessment of single OCPs showed that the RQ values of HCB at B9, B14, B16, and B17 were 1.27, 1.22, 1.05, and 1.08, and the RQ values of endosulfan I at B8 and B12 were 1.54, and 3.39, respectively, indicating high ecological risks (Table S5).

#### 3.3.2. Health Risk Assessment

**Figure 5:**
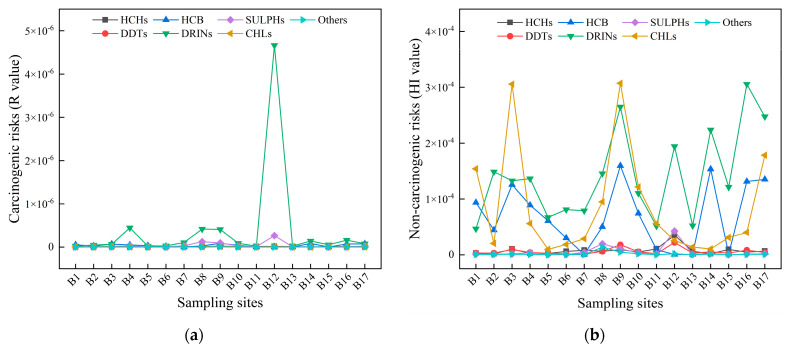
The carcinogenic risk (**a**) and non−carcinogenic risk (**b**) of OCPs in water of the Beiluo Basin.

Figure 5 shows the health risk assessment results of OCPs in water of the Beiluo River. During the assessment, it was found that the OCP intake generated by the drinking water exposure route was much greater than the OCP intake generated by the skin contact exposure route, and the HI value was generally greater than the R-value. Except for the DRINs in B12, the R-value of the carcinogenic risk assessment results of OCPs in other water bodies was lower than 1 × 10^−6^, and the R-value of DRINs in B12 was 4.66 × 10^−6^, not exceeding 1 × 10^−4^ (Figure 5a). In general, the residual amount of OCPs in Beiluo River water samples is not enough to pose a carcinogenic health risk to humans. However, DRINs have a potential carcinogenic risk with an R-value of more than 1 × 10^−6^ at B12. The non-carcinogenic risk assessment results of OCPs in Beiluo River showed that the HI value range was 3.20 × 10^−7^–3.06 × 10^−4^ (Figure 5b). The results were all lower than the USEPA’s recommended baseline value one, indicating that OCPs in the Beiluo River water body did not produce significant non-carcinogenic health effects on humans.

## 4. Discussion

### 4.1. Residual status of OCPs in Water

In this study, OCP concentrations in water of the Beiluo River were monitored, and the results showed that DRINs’ mean concentration was the highest in the river. This is because DRINs, compared with other OCPs, have a higher soil adsorption coefficient and accumulate more easily [49]. However, DRINs remaining in the soil will enter the river with rainwater runoff and increase river concentrations [1]. The rapid development of agriculture in the middle stream and the extensive use of SULPHs and DRINs have resulted in high levels of OCP pollution in the region [49,50]. OCP content in B13 decreased significantly compared with that in B12, possibly due to heavy rainfall in the Beiluo River region in June. The pollutants were diluted under the erosion of heavy rain, and the complex and changeable landforms in this region led to an uneven distribution of pollutant concentrations, resulting in a sharp decline in concentration [51].

Previously, Zhao et al. collected 15 water samples in densely populated areas of the Yellow River Basin in September 2018 [52], and the mean concentration of 14 OCPs in water was 0.748 ng L^−1^. Compared with the results of OCPs in the water of the Beiluo River, the OCP concentration measured by Zhao et al. was relatively low, which may be due to the fact that, considering human health and drinking water safety, there are fewer factories and farms in densely populated areas as well as fewer pollution sources. In addition, Fosu-Mensah et al. monitored OCPs in drinking water sources near cacao farms in Ghana [53] and found that the concentration range of OCPs was nd−160 ng L^−1^ at 0–15 m away from the farm. However, OCPs could not be detected beyond 30 m away from the farm. This further verifies the significant influence of agricultural or industrial activities on OCP pollution. The collection sites of water samples in this study may be close to specific pollution sources, so it can be inferred that there is a high degree of OCP pollution in the water of the Beiluo River. Table 1 lists the concentration data of OCPs in rivers or marine systems from other studies at home and abroad. Through comparison, it was found that the concentration of OCPs in Beiluo River water is at a moderate level. Other studies have also found a positive correlation between OCP pollution levels and industrial or agricultural areas: Hangzhou Bay (2.26–102.07 ng L^−1^) [1], Yongding River (0.08–83.58 ng L^−1^) [54], and Taihu Lake (80.95–376.03 ng L^−1^) [55]. In addition, in the water samples of Hangzhou Bay, there was a significant difference in OCP concentration between summer (2.62–10.11 ng L^−1^) and autumn (11.86–102.07 ng L^−1^), which was mainly related to the seasonal variation of the tidal range of Hangzhou Bay [1,56]. Therefore, further monitoring of OCP concentration in autumn water samples from the Beiluo River should be considered to explore the influence of natural conditions, such as river velocity and riparian topography on the migration and distribution of OCPs.

### 4.2. Source Analysis of OCPs in Water

The results of the source apportionment of OCPs show that our country has obtained some effect by prohibiting the production and use of OCPs [62]. The Loess Plateau is an essential area in China’s western development and regional economic and social development. Energy, the chemical industry, and other fields have developed rapidly. The industrial system based on coal, oil, and natural gas has built the main engine of economic and social development [63,64]. The middle and upper reaches of the Beiluo River are home to numerous chemical industries, steel mills, and refineries, and oil production and transportation is one of the most important economic activities in the region [65,66]. However, these activities have produced a large amount of toxic waste and spilled oil, and the water bodies are seriously polluted. At the same time, the heavy use of pesticides and fertilizers also has an impact on the watershed environment. Studies have shown that OCP pollution may be caused by oil exploration and intensive agricultural activities, as well as the use of insecticides [67]. In addition to the potential sources described in Section 3.2, OCP pollution in the Beiluo River Basin may also be caused by long-distance atmospheric migration [68]. Some studies showed that DRINs (41 ng kg^−1^) were detected in the non-cultivated soil of the Tibetan Plateau, which may be due to the large amount of DRINs used in India which were then transported and deposited into the Tibetan Plateau soil through the atmosphere [69]. Therefore, it is necessary to investigate and monitor the distribution of OCP content in the atmosphere in this region in the future, so as to understand the possible sources of OCPs more comprehensively.

### 4.3. Risk Analysis for OCPs in Water

In this study, ecological risk assessment of OCPs in water from the Beiluo River was carried out, and the results showed that the area with the highest ecological risk was located in the middle stream of the Beiluo River, mainly because agriculture occupies a large proportion of this region and the use of pesticides is much higher than in other regions [13]. HCB has been in the spotlight because of its wide range of occurrence and high environmental risks. It was one of the first persistent organic pollutants (POPs) listed by the Stockholm Convention. Most countries, including China, banned the use of HCB after 2009 [70,71]. According to previous studies, HCB exhibits long-distance migration and bioaccumulation properties, because it usually interacts with hydroxyl radicals, and its half-life in the atmosphere is 7.7–14 years [72], suggesting that even trace amounts of HCB will still have a significant impact on aquatic systems. Endosulfan has been found to be very harmful to aquatic life, and in high concentrations it can even cause the death of large fish [41]. The results of source analysis showed that endosulfan was recently imported into the middle reaches of the Beiluo River, indicating that illegal use of endosulfan may have occurred. Thus, relevant departments should further strengthen monitoring and control of HCB and endosulfan pollution in the middle and downstream of the Beiluo River.

We conducted an OCP health risk assessment in the Beiluo River Basin and found that DRINs in B12 have a potential carcinogenic risk. It is known that DRINs pose a certain degree of threat to human health around the world. After entering the human body through different exposure routes, DRINs accumulate because the human body cannot metabolize them [73]. DRINs are immunogenic in humans and lead to dopaminergic neurodegeneration, which causes chemically immunohemolytic anemia, Parkinson’s disease, or liver dysfunction [74,75]. Therefore, continuous monitoring and risk assessment of DRINs should be carried out in the future. If necessary, physical and chemical methods [76] or microbial metabolism [77,78] can be used to degrade residual DRINs to ensure and control the safety of water use [55].

## 5. Conclusions

This study analyzed the pollution distribution characteristics, source identification, and potential ecological health risks of OCPs in surface water of the Beiluo River. The results of this study will provide ideas for the formulation and implementation of an OCP emission reduction strategy in China and the improvement of water quality in the Beiluo River Basin. In addition, the results provide a reference for establishing a global baseline of POP levels and predicting changes in POP concentrations. In view of the ecological health risks from OCPs in the middle and lower reaches of the Beiluo River Basin, the pollution status of the basin should be monitored regularly, and the feasibility and importance of risk assessment of OCPs in the Beiluo River Basin should be emphasized. In addition, in the follow-up study of organic pollution in this watershed, comprehensive monitoring in different seasons should be carried out to reveal the changing pattern of OCPs.

## Figures and Tables

**Table 1:** Comparisons of OCPs (ng∙L^−1^) in water from different regions.

Location	OCP Range (ng L^−1^)	Mean (ng L^−1^)	Reference
Beiluo River, China	1.76–32.57	7.23	This study
Yellow River, China	0.231–2.22	0.748	[52]
Yongding River, China	0.08–83.58	7.92	[54]
Taihu Lake, China	80.95–376.03	-	[55]
Hangzhou Bay (Summer), China	2.62–10.11	4.99	[1]
Hangzhou Bay (Autumn), China	11.86–102.07	56.32	[1]
Krishna River, India	1.08–5.44	-	[57]
Brong–Ahafo Region, Ghana	nd *–160	-	[53]
Volturno River, Italy	0.93–8.66	-	[58]
In and Outside Rosetta Branch Estuary, Egypt	1.92–4.00	-	[59]
Akaki River and Aba Samuel Reservoir, Ethiopia	5.33–30.58	11.39	[60]
Jaguaribe River, Brazil	5.09–154.43	-	[61]

* nd = not detected.

## Data Availability

Not applicable.

## Supplementary Materials

The following supporting information can be downloaded at: https://www.mdpi.com/article/10.3390/toxics11060496/s1, Table S1: Parameters of health risk assessment model; Table S2: Distribution of OCPs in water of the Beiluo River (ng L^−1^); Table S3: Descriptive statistics of OCP concentrations in water of the Beiluo River (ng L^−1^); Table S4: The ratio distribution of chlordane isomers in water of the Beiluo River; Table S5: The ecological risk of OCPs in water of the Beiluo River.
