# Supplementary material for: Distribution, Sources, and Risk Assessment of Organochlorine Pesticides in Water from Beiluo River, Loess Plateau, China
Source: Toxics. 2023 May 31;11(6):496. doi: 10.3390/toxics11060496 (PMC10302601; doi:10.3390/toxics11060496)
Supplement: Supplementary file 1 [file toxics-11-00496-s001.zip › toxics-2374985-supplementary.pdf]

toxics

MDPI

Supplementary data

Distribution, Sources, and Risk Assessment of Organochlorine Pesticides in Water from Beiluo River, Loess Plateau, China

Jipu Guo [1], Wenwu Chen [2], Menglei Wu [3], Chengkai Qu [4], Haotian Sun [2] and Jiahua Guo [2,*]

Table S1. Parameters of health risk assessment model.

Parameter	Description	Unit	Value
IR	Daily intake of water	L/d	2
EF	Exposure frequency	d/a	365
ED	Exposure duration	a	30
BW	Average body weight	kg	60
AT	Average exposure time	d	Non-carcinogenic ED*365<br>Carcinogenic 25550
k	Dermal permeability coefficient	cm/h	0.001
SA	Exposed skin area	cm^2	16600
FE	Body bath frequency	n/d	0.3
f	Intestinal absorption ratio	Unitless	1
τ	Delay time	h	1
TE	Dermal contact time	h	0.4
SF	Cancer slope factor	(kg·d)/mg	Chemical-specific
RfD	Reference dose	mg/(kg·d)	Chemical-specific

Table S2. Distribution of OCPs in water of the Beiluo River (ng L^{-1}).

Substance	B1	B2	B3	B4	B5	B6	B7	B8	B9
α-HCH	6.28×10^{-2}	3.03×10^{-2}	1.91×10^{-1}	3.65×10^{-2}	3.35×10^{-2}	1.13×10^{-1}	1.18×10^{-1}	1.07×10^{-1}	1.55×10^{-1}
β-HCH	1.33×10^{-1}	3.16×10^{-2}	1.26×10^{-1}	7.29×10^{-2}	6.38×10^{-2}	2.00×10^{-1}	2.76×10^{-1}	1.39×10^{-1}	8.42×10^{-2}
γ-HCH	2.23×10^{-2}	1.51×10^{-2}	8.21×10^{-2}	2.10×10^{-2}	1.90×10^{-2}	4.81×10^{-2}	6.45×10^{-2}	5.02×10^{-2}	7.38×10^{-2}
δ-HCH	6.08×10^{-2}	1.35×10^{-2}	2.77×10^{-2}	5.27×10^{-2}	9.06×10^{-3}	3.34×10^{-2}	3.89×10^{-2}	1.17×10^{-1}	2.06×10^{-2}
o,p′-DDD	1.25×10^{-2}	5.88×10^{-3}	7.10×10^{-3}	2.66×10^{-2}	1.61×10^{-3}	4.37×10^{-3}	5.82×10^{-3}	8.75×10^{-3}	9.13×10^{-2}
p,p′-DDD	6.38×10^{-3}	9.98×10^{-3}	5.09×10^{-3}	1.27×10^{-2}	4.72×10^{-3}	4.99×10^{-3}	4.05×10^{-3}	2.19×10^{-2}	1.12×10^{-1}
o,p′-DDT	1.80×10^{-2}	1.83×10^{-2}	2.99×10^{-2}	2.79×10^{-2}	5.71×10^{-3}	9.18×10^{-3}	1.70×10^{-2}	3.06×10^{-2}	1.82×10^{-2}
p,p′-DDT	2.75×10^{-2}	2.29×10^{-2}	1.23×10^{-1}	3.29×10^{-2}	2.80×10^{-2}	1.09×10^{-2}	3.18×10^{-3}	3.85×10^{-2}	1.43×10^{-1}
o,p′-DDE	1.30×10^{-3}	1.95×10^{-3}	5.57×10^{-3}	1.17×10^{-3}	5.01×10^{-4}	1.06×10^{-3}	4.11×10^{-3}	8.81×10^{-3}	1.81×10^{-2}
p,p′-DDE	7.52×10^{-1}	8.69×10^{-1}	6.55×10^{-1}	1.13×10^{0}	4.57×10^{-1}	5.05×10^{-1}	6.24×10^{-1}	2.04×10^{0}	4.10×10^{0}
HCB	2.24×10^{0}	1.06×10^{0}	3.00×10^{0}	2.12×10^{0}	1.46×10^{0}	7.16×10^{-1}	1.48×10^{-2}	1.20×10^{0}	3.81×10^{0}
aldrin	2.68×10^{-2}	1.19×10^{-1}	1.02×10^{-1}	9.04×10^{-2}	5.55×10^{-2}	6.47×10^{-2}	6.04×10^{-2}	1.18×10^{-1}	7.90×10^{-2}
dieldrin	1.94×10^{-2}	4.73×10^{-2}	1.20×10^{-1}	1.79×10^{0}	3.48×10^{-2}	2.92×10^{-2}	3.53×10^{-1}	1.56×10^{0}	1.21×10^{0}
endrin	1.46×10^{-1}	1.39×10^{-1}	1.61×10^{-1}	3.14×10^{-1}	4.56×10^{-2}	7.70×10^{-2}	9.84×10^{-2}	1.10×10^{-1}	1.55×10^{0}
endrin aldehyde	9.58×10^{-2}	6.98×10^{-2}	2.68×10^{-1}	6.77×10^{-2}	4.53×10^{-3}	7.51×10^{-2}	4.10×10^{-3}	2.57×10^{-1}	9.80×10^{-2}
endrin ketone	1.36×10^{-1}	3.59×10^{-2}	3.46×10^{-2}	1.29×10^{-1}	1.25×10^{-2}	2.72×10^{-2}	2.17×10^{-1}	7.05×10^{-1}	2.24×10^{0}
endosulfan I	1.28×10^{-1}	1.93×10^{-1}	1.32×10^{-1}	5.67×10^{-1}	2.61×10^{-2}	5.46×10^{-2}	6.95×10^{-1}	3.08×10^{0}	8.22×10^{-1}
endosulfan II	1.30×10^{-1}	1.28×10^{-1}	3.85×10^{-1}	3.19×10^{-1}	1.60×10^{-1}	9.56×10^{-2}	3.58×10^{-1}	1.10×10^{0}	2.02×10^{0}
endosulfan sulfate	1.30×10^{-2}	5.02×10^{-3}	3.15×10^{-2}	2.93×10^{-2}	5.76×10^{-3}	2.99×10^{-3}	2.58×10^{-2}	8.73×10^{-2}	5.01×10^{-1}
cis-Chlordane	3.82×10^{-2}	4.03×10^{-3}	1.10×10^{-2}	1.77×10^{-2}	4.88×10^{-3}	2.43×10^{-2}	1.78×10^{-2}	5.11×10^{-2}	3.36×10^{-1}
trans-Chlordane	2.31×10^{-3}	8.31×10^{-3}	7.34×10^{-2}	2.03×10^{-2}	1.17×10^{-2}	1.26×10^{-2}	1.33×10^{-2}	5.64×10^{-2}	3.07×10^{-2}
heptachlor	1.12×10^{-2}	6.33×10^{-3}	1.40×10^{-2}	1.11×10^{-2}	6.52×10^{-4}	8.96×10^{-3}	1.57×10^{-2}	5.59×10^{-2}	2.38×10^{-2}
heptachlor epoxide isomer B	5.85×10^{-2}	7.40×10^{-3}	1.16×10^{-1}	2.05×10^{-2}	3.35×10^{-3}	5.96×10^{-3}	9.92×10^{-3}	3.26×10^{-2}	1.09×10^{-1}

methoxychlor	1.49×10^{-2}	1.75×10^{-2}	1.39×10^{-2}	3.55×10^{-2}	7.17×10^{-3}	1.32×10^{-2}	3.55×10^{-2}	2.91×10^{-2}	1.68×10^{-1}
mirex	4.84×10^{-3}	2.69×10^{-3}	6.29×10^{-3}	2.28×10^{-3}	1.42×10^{-3}	1.83×10^{-3}	7.55×10^{-4}	8.10×10^{-2}	2.26×10^{-2}

**Substance**	B10	B11	B12	B13	B14	B15	B16	B17
α-HCH	4.74×10^{-2}	1.08×10^{-1}	1.89×10^{-1}	1.13×10^{-1}	4.92×10^{-2}	1.44×10^{-1}	1.18×10^{-1}	1.64×1[illegible]
β-HCH	9.76×10^{-2}	2.53×10^{-1}	4.50×10^{-2}	2.09×10^{-1}	6.35×10^{-2}	2.18×10^{-1}	1.23×10^{-1}	1.64×1[illegible]
γ-HCH	4.14×10^{-2}	8.55×10^{-2}	3.03×10^{-1}	4.86×10^{-2}	1.43×10^{-2}	7.73×10^{-2}	3.64×10^{-2}	5.14×1[illegible]
δ-HCH	9.54×10^{-2}	3.84×10^{-2}	7.66×10^{-2}	2.75×10^{-2}	1.64×10^{-2}	3.37×10^{-2}	2.64×10^{-2}	3.40×1[illegible]
o,p′-DDD	1.46×10^{-2}	7.64×10^{-3}	3.66×10^{-1}	2.03×10^{-3}	2.21×10^{-3}	3.36×10^{-3}	1.32×10^{-2}	4.47×1[illegible]
p,p′-DDD	6.70×10^{-3}	8.51×10^{-3}	3.30×10^{-2}	8.58×10^{-3}	2.44×10^{-2}	2.42×10^{-2}	1.71×10^{-2}	1.33×1[illegible]
o,p′-DDT	1.14×10^{-2}	9.07×10^{-3}	3.73×10^{-2}	1.31×10^{-2}	2.06×10^{-2}	9.13×10^{-3}	2.57×10^{-2}	1.45×1[illegible]
p,p′-DDT	3.59×10^{-2}	1.11×10^{-2}	2.93×10^{-1}	2.20×10^{-2}	3.74×10^{-2}	2.91×10^{-2}	5.46×10^{-2}	2.23×1[illegible]
o,p′-DDE	3.18×10^{-4}	7.74×10^{-4}	7.66×10^{-3}	1.42×10^{-3}	4.85×10^{-3}	1.63×10^{-3}	1.02×10^{-2}	4.76×1[illegible]
p,p′-DDE	1.81×10^{0}	7.56×10^{-1}	7.32×10^{-1}	8.30×10^{-1}	1.47×10^{0}	6.28×10^{-1}	2.57×10^{0}	1.56×1[illegible]
HCB	1.77×10^{0}	2.18×10^{-1}	1.48×10^{-2}	1.48×10^{-2}	3.67×10^{0}	2.59×10^{-2}	3.14×10^{0}	3.23×1[illegible]
aldrin	8.30×10^{-2}	4.23×10^{-2}	1.42×10^{-1}	3.24×10^{-2}	1.74×10^{-1}	9.89×10^{-2}	2.21×10^{-1}	2.13×1[illegible]
dieldrin	2.23×10^{-1}	2.67×10^{-2}	2.01×10^{1}	3.31×10^{-2}	3.22×10^{-1}	9.28×10^{-2}	3.41×10^{-1}	6.25×1[illegible]
endrin	1.52×10^{-1}	3.63×10^{-2}	3.01×10^{-1}	1.43×10^{-1}	2.52×10^{-1}	9.51×10^{-2}	5.23×10^{-1}	7.89×1[illegible]
endrin aldehyde	3.58×10^{-1}	6.36×10^{-2}	7.83×10^{-2}	2.74×10^{-2}	9.82×10^{-2}	9.39×10^{-2}	3.11×10^{-1}	2.14×1[illegible]
endrin ketone	3.77×10^{-2}	9.04×10^{-2}	3.07×10^{-1}	6.09×10^{-2}	4.47×10^{-1}	6.38×10^{-2}	1.48×10^{-1}	6.39×1[illegible]
endosulfan I	4.94×10^{-1}	2.64×10^{-1}	6.78×10^{0}	4.16×10^{-2}	2.12×10^{-1}	5.19×10^{-2}	1.56×10^{-1}	1.25×1[illegible]
endosulfan II	3.65×10^{-1}	4.75×10^{-2}	2.29×10^{0}	4.93×10^{-2}	2.76×10^{-2}	1.34×10^{-2}	4.51×10^{-2}	6.46×1[illegible]
endosulfan sulfate	1.33×10^{-2}	1.27×10^{-2}	1.79×10^{-1}	5.98×10^{-2}	2.29×10^{-1}	1.48×10^{-3}	6.21×10^{-2}	2.10×1[illegible]
cis-Chlordane	7.67×10^{-2}	4.92×10^{-3}	1.59×10^{-1}	1.00×10^{-3}	1.38×10^{-3}	1.18×10^{-2}	8.45×10^{-2}	7.80×1[illegible]
trans-Chlordane	3.53×10^{-2}	3.31×10^{-2}	9.92×10^{-3}	6.01×10^{-3}	2.93×10^{-2}	9.11×10^{-3}	1.69×10^{-1}	7.79×1[illegible]
heptachlor	1.49×10^{-2}	4.37×10^{-3}	1.10×10^{-1}	4.91×10^{-3}	1.27×10^{-2}	1.28×10^{-2}	1.48×10^{-2}	3.70×1[illegible]
heptachlor epoxide isomer B	4.39×10^{-2}	2.07×10^{-2}	2.67×10^{-3}	5.11×10^{-3}	2.91×10^{-3}	1.12×10^{-2}	8.59×10^{-3}	6.51×1[illegible]
methoxychlor	2.43×10^{-2}	1.68×10^{-2}	1.76×10^{-2}	1.10×10^{-2}	1.02×10^{-1}	5.72×10^{-3}	1.74×10^{-2}	2.42×1[illegible]
mirex	9.67×10^{-3}	8.55×10^{-4}	1.01×10^{-2}	1.24×10^{-3}	2.15×10^{-3}	2.30×10^{-3}	4.11×10^{-3}	5.68×1[illegible]

Table S3. Descriptive statistics of OCPs concentrations in water of the Beiluo River (ng L^{-1}).

Substance	MDLs (ng L^{-1})	Mean (ng L^{-1})	Range (ng L^{-1})	SD (ng L^{-1})	CV
α-HCH	0.0086	0.10	0.030-0.19	0.054	0.51
β-HCH	0.015	0.14	0.032-0.28	0.074	0.55
γ-HCH	0.020	0.062	0.014-0.30	0.067	1.073
δ-HCH	0.12	0.042	0.0091-0.12	0.030	0.70
**HCHs**		**0.34**	**0.090-0.61**	**0.15**	**0.42**
o,p′-DDD	0.0083	0.034	0.0016-0.37	0.088	2.59
p,p′-DDD	0.011	0.019	0.0041-0.11	0.026	1.37
o,p′-DDT	0.041	0.019	0.0057-0.037	0.0090	0.49
p,p′-DDT	0.017	0.055	0.0032-0.29	0.072	1.30
o,p′-DDE	0.0072	0.0044	0.00032-0.018	0.0047	1.075
p,p′-DDE	0.56	1.26	0.46-4.1	0.95	0.75
**DDTs**		**1.39**	**0.50-4.48**	**1.0060**	**0.72**
**HCB**	0.030	**1.63**	**0.015-3.81**	**1.37**	**0.84**
aldrin	0.022	0.10	0.027-0.22	0.058	0.57
dieldrin	0.11	1.55	0.019-20.08	4.81	3.10

endrin	0.13	0.25	0.036-1.55	0.36	1.44
endrin aldehyde	0.010	0.13	0.0041-0.36	0.11	0.85
endrin ketone	0.38	0.28	0.012-2.24	0.54	1.92
**DRINs**		**2.31**	**0.15-20.91**	**4.96**	**2.15**
endosulfan I	0.36	0.81	0.026-6.78	1.70	2.090
endosulfan II	0.037	0.48	0.013-2.29	0.69	1.43
endosulfan sulfate	0.11	0.075	0.0015-0.5	0.13	1.69
**SULPHs**		**1.37**	**0.067-9.25**	**2.34**	**1.71**
cis-Chlordane	0.031	0.054	0.0010-0.34	0.084	1.55
trans-Chlordane	0.032	0.035	0.0023-0.17	0.041	1.18
heptachlor	0.019	0.019	0.00065-0.11	0.026	1.38
heptachlor epoxide isomer B	0.022	0.031	0.0027-0.12	0.036	1.18
**CHLs**		**0.14**	**0.017-0.50**	**0.13**	**0.93**
methoxychlor	0.20	0.033	0.0057-0.17	0.041	1.26
mirex	0.0043	0.0094	0.00075-0.081	0.019	2.044
**Others**		**0.042**	**0.0080-0.19**	**0.048**	**1.15**
**ΣOCPs**		**7.23**	**1.76-32.57**	**7.72**	**1.069**

Table. S4. The ratio distribution of chlordane isomers in water of the Beiluo River

	B1	B2	B3	B4	B5	B6	B7	B8	B9	B10	B11	B12	B13	B14	B15	B16	B17
**cis-chlordane (CC)**	3.82×10^{-2}	4.03×10^{-3}	1.10×10^{-2}	1.77×10^{-2}	4.88×10^{-3}	2.43×10^{-2}	1.78×10^{-2}	5.11×10^{-2}	3.36×10^{-1}	7.67×10^{-2}	4.92×10^{-3}	1.59×10^{-1}	1.00×10^{-3}	1.38×10^{-3}	1.18×10^{-2}	8.45×10^{-2}	7.80×10^{-2}
**trans-chlordane (TC)**	2.31×10^{-3}	8.31×10^{-3}	7.34×10^{-2}	2.03×10^{-2}	1.17×10^{-2}	1.26×10^{-2}	1.33×10^{-2}	5.64×10^{-2}	3.07×10^{-2}	3.53×10^{-2}	3.31×10^{-2}	9.92×10^{-3}	6.01×10^{-3}	2.93×10^{-2}	9.11×10^{-3}	1.69×10^{-1}	7.79×10^{-2}
**CC/TC**	1.65×10^{1}	4.85×10^{-1}	1.50×10^{-1}	8.74×10^{-1}	4.16×10^{-1}	1.93×10^{0}	1.34×10^{0}	9.07×10^{-1}	1.09×10^{1}	2.17×10^{0}	1.49×10^{-1}	1.60×10^{1}	1.67×10^{-1}	4.71×10^{-2}	1.29×10^{0}	4.99×10^{-1}	1.00×10^{0}

Table S5. The ecological risk of OCPs in water of the Beiluo River.

Substance	PNEC	B1	B2	B3	B4	B5	B6	B7	B8	B9
α-HCH	370	1.70×10^{-4}	8.18×10^{-5}	5.17×10^{-4}	9.88×10^{-5}	9.04×10^{-5}	3.05×10^{-4}	3.18×10^{-4}	2.88×10^{-4}	4.18×10^{-4}
β-HCH	3140	4.22×10^{-5}	1.01×10^{-5}	4.01×10^{-5}	2.32×10^{-5}	2.03×10^{-5}	6.37×10^{-5}	8.77×10^{-5}	4.43×10^{-5}	2.68×10^{-5}
γ-HCH	2.9	7.69×10^{-3}	5.20×10^{-3}	2.83×10^{-2}	7.23×10^{-3}	6.55×10^{-3}	1.66×10^{-2}	2.22×10^{-2}	1.73×10^{-2}	2.55×10^{-2}
δ-HCH	1580	3.85×10^{-5}	8.54×10^{-6}	1.75×10^{-5}	3.33×10^{-5}	5.73×10^{-6}	2.12×10^{-5}	2.46×10^{-5}	7.40×10^{-5}	1.30×10^{-5}
o,p′-DDD	37.9	3.30×10^{-4}	1.55×10^{-4}	1.87×10^{-4}	7.02×10^{-4}	4.24×10^{-5}	1.15×10^{-4}	1.54×10^{-4}	2.31×10^{-4}	2.41×10^{-3}
p,p′-DDD	9	7.09×10^{-4}	1.11×10^{-3}	5.66×10^{-4}	1.42×10^{-3}	5.25×10^{-4}	5.55×10^{-4}	4.50×10^{-4}	2.43×10^{-3}	1.25×10^{-2}
o,p′-DDT	30	6.01×10^{-4}	6.10×10^{-4}	9.96×10^{-4}	9.30×10^{-4}	1.90×10^{-4}	3.06×10^{-4}	5.68×10^{-4}	1.02×10^{-3}	6.06×10^{-4}
p,p′-DDT	9	3.06×10^{-3}	2.54×10^{-3}	1.37×10^{-2}	3.65×10^{-3}	3.11×10^{-3}	1.22×10^{-3}	3.53×10^{-4}	4.28×10^{-3}	1.59×10^{-2}
o,p′-DDE	15	8.64×10^{-5}	1.30×10^{-4}	3.71×10^{-4}	7.78×10^{-5}	3.34×10^{-5}	7.06×10^{-5}	2.74×10^{-4}	5.87×10^{-4}	1.21×10^{-3}
p,p′-DDE	53.5	1.41×10^{-2}	1.62×10^{-2}	1.22×10^{-2}	2.12×10^{-2}	8.54×10^{-3}	9.45×10^{-3}	1.17×10^{-2}	3.81×10^{-2}	7.66×10^{-2}
HCB	3	7.45×10^{-1}	3.54×10^{-1}	9.99×10^{-1}	7.07×10^{-1}	4.85×10^{-1}	2.39×10^{-1}	4.94×10^{-3}	4.00×10^{-1}	**1.27×10^{0}**
aldrin	10	2.68×10^{-3}	1.19×10^{-2}	1.02×10^{-2}	9.04×10^{-3}	5.55×10^{-3}	6.47×10^{-3}	6.04×10^{-3}	1.18×10^{-2}	7.90×10^{-3}
dieldrin	50	3.89×10^{-4}	9.47×10^{-4}	2.40×10^{-3}	3.58×10^{-2}	6.96×10^{-4}	5.84×10^{-4}	7.06×10^{-3}	3.12×10^{-2}	2.43×10^{-2}

endrin	4.2	3.48×10^{-2}	3.30×10^{-2}	3.82×10^{-2}	7.47×10^{-2}	1.09×10^{-2}	1.83×10^{-2}	2.34×10^{-2}	2.63×10^{-2}	3.69×10^{-1}
endrin aldehyde	42	2.28×10^{-3}	1.66×10^{-3}	6.38×10^{-3}	1.61×10^{-3}	1.08×10^{-4}	1.79×10^{-3}	9.75×10^{-5}	6.12×10^{-3}	2.33×10^{-3}
endrin ketone	42	3.24×10^{-3}	8.55×10^{-4}	8.25×10^{-4}	3.06×10^{-3}	2.97×10^{-4}	6.47×10^{-4}	5.17×10^{-3}	1.68×10^{-2}	5.33×10^{-2}
endosulfan I	2	6.41×10^{-2}	9.63×10^{-2}	6.60×10^{-2}	2.84×10^{-1}	1.30×10^{-2}	2.73×10^{-2}	3.48×10^{-1}	**1.54×10^{0}**	4.11×10^{-1}
endosulfan II	4.9	2.65×10^{-2}	2.62×10^{-2}	7.87×10^{-2}	6.51×10^{-2}	3.27×10^{-2}	1.95×10^{-2}	7.31×10^{-2}	2.25×10^{-1}	4.12×10^{-1}
endosulfan sulfate	20	6.51×10^{-4}	2.51×10^{-4}	1.57×10^{-3}	1.46×10^{-3}	2.88×10^{-4}	1.50×10^{-4}	1.29×10^{-3}	4.36×10^{-3}	2.50×10^{-2}
cis-Chlordane	90	4.24×10^{-4}	4.48×10^{-5}	1.22×10^{-4}	1.97×10^{-4}	5.42×10^{-5}	2.69×10^{-4}	1.98×10^{-4}	5.68×10^{-4}	3.74×10^{-3}
trans-Chlordane	12	1.92×10^{-4}	6.92×10^{-4}	6.12×10^{-3}	1.69×10^{-3}	9.78×10^{-4}	1.05×10^{-3}	1.11×10^{-3}	4.70×10^{-3}	2.56×10^{-3}
heptachlor	38.1	2.94×10^{-4}	1.66×10^{-4}	3.67×10^{-4}	2.90×10^{-4}	1.71×10^{-5}	2.35×10^{-4}	4.12×10^{-4}	1.47×10^{-3}	6.23×10^{-4}
heptachlor epoxide isomer B	84	6.96×10^{-4}	8.81×10^{-5}	1.38×10^{-3}	2.44×10^{-4}	3.99×10^{-5}	7.10×10^{-5}	1.18×10^{-4}	3.88×10^{-4}	1.30×10^{-3}
methoxychlor	150	9.93×10^{-5}	1.17×10^{-4}	9.26×10^{-5}	2.37×10^{-4}	4.78×10^{-5}	8.83×10^{-5}	2.37×10^{-4}	1.94×10^{-4}	1.12×10^{-3}
mirex	1000	4.84×10^{-6}	2.69×10^{-6}	6.29×10^{-6}	2.28×10^{-6}	1.42×10^{-6}	1.83×10^{-6}	7.55×10^{-7}	8.10×10^{-5}	2.26×10^{-5}
**ΣOCPs**		9.08×10^{-1}	5.52×10^{-1}	**1.27×10^{0}**	**1.22×10^{0}**	5.69×10^{-1}	3.44×10^{-1}	5.07×10^{-1}	**2.33×10^{0}**	**2.72×10^{0}**

Substance	PNEC	B10	B11	B12	B13	B14	B15	B16	B17
α-HCH	370	1.28×10^{-4}	2.91×10^{-4}	5.12×10^{-4}	3.05×10^{-4}	1.33×10^{-4}	3.89×10^{-4}	3.20×10^{-4}	4.44×10^{-4}
β-HCH	3140	3.11×10^{-5}	8.05×10^{-5}	1.43×10^{-5}	6.66×10^{-5}	2.02×10^{-5}	6.93×10^{-5}	3.92×10^{-5}	5.21×10^{-5}
γ-HCH	2.9	1.43×10^{-2}	2.95×10^{-2}	1.05×10^{-1}	1.68×10^{-2}	4.91×10^{-3}	2.66×10^{-2}	1.26×10^{-2}	1.77×10^{-2}
δ-HCH	1580	6.04×10^{-5}	2.43×10^{-5}	4.85×10^{-5}	1.74×10^{-5}	1.04×10^{-5}	2.14×10^{-5}	1.67×10^{-5}	2.15×10^{-5}
o,p′-DDD	37.9	3.85×10^{-4}	2.02×10^{-4}	9.65×10^{-3}	5.35×10^{-5}	5.84×10^{-5}	8.85×10^{-5}	3.49×10^{-4}	1.18×10^{-4}
p,p′-DDD	9	7.44×10^{-4}	9.46×10^{-4}	3.67×10^{-3}	9.53×10^{-4}	2.72×10^{-3}	2.69×10^{-3}	1.90×10^{-3}	1.48×10^{-3}
o,p′-DDT	30	3.81×10^{-4}	3.02×10^{-4}	1.24×10^{-3}	4.37×10^{-4}	6.86×10^{-4}	3.04×10^{-4}	8.56×10^{-4}	4.83×10^{-4}
p,p′-DDT	9	3.99×10^{-3}	1.23×10^{-3}	3.26×10^{-2}	2.44×10^{-3}	4.16×10^{-3}	3.23×10^{-3}	6.07×10^{-3}	2.47×10^{-3}
o,p′-DDE	15	2.12×10^{-5}	5.16×10^{-5}	5.11×10^{-4}	9.47×10^{-5}	3.23×10^{-4}	1.08×10^{-4}	6.81×10^{-4}	3.17×10^{-4}
p,p′-DDE	53.5	3.38×10^{-2}	1.41×10^{-2}	1.37×10^{-2}	1.55×10^{-2}	2.75×10^{-2}	1.17×10^{-2}	4.80×10^{-2}	2.91×10^{-2}
HCB	3	5.90×10^{-1}	7.27×10^{-2}	4.94×10^{-3}	4.94×10^{-3}	**1.22×10^{0}**	8.63×10^{-3}	**1.05×10^{0}**	**1.08×10^{0}**
aldrin	10	8.30×10^{-3}	4.23×10^{-3}	1.42×10^{-2}	3.24×10^{-3}	1.74×10^{-2}	9.89×10^{-3}	2.21×10^{-2}	2.13×10^{-2}
dieldrin	50	4.45×10^{-3}	5.34×10^{-4}	4.02×10^{-1}	6.62×10^{-4}	6.45×10^{-3}	1.86×10^{-3}	6.83×10^{-3}	1.25×10^{-3}
endrin	4.2	3.61×10^{-2}	8.63×10^{-3}	7.16×10^{-2}	3.41×10^{-2}	6.01×10^{-2}	2.26×10^{-2}	1.25×10^{-1}	1.88×10^{-2}
endrin aldehyde	42	8.52×10^{-3}	1.51×10^{-3}	1.87×10^{-3}	6.52×10^{-4}	2.34×10^{-3}	2.24×10^{-3}	7.41×10^{-3}	5.09×10^{-3}
endrin ketone	42	8.98×10^{-4}	2.15×10^{-3}	7.32×10^{-3}	1.45×10^{-3}	1.06×10^{-2}	1.52×10^{-3}	3.52×10^{-3}	1.52×10^{-3}
endosulfan I	2	2.47×10^{-1}	1.32×10^{-1}	**3.39×10^{0}**	2.08×10^{-2}	1.06×10^{-1}	2.59×10^{-2}	7.80×10^{-2}	6.24×10^{-2}
endosulfan II	4.9	7.44×10^{-2}	9.70×10^{-3}	4.67×10^{-1}	1.01×10^{-2}	5.64×10^{-3}	2.73×10^{-3}	9.20×10^{-3}	1.32×10^{-1}
endosulfan sulfate	20	6.63×10^{-4}	6.33×10^{-4}	8.95×10^{-3}	2.99×10^{-3}	1.14×10^{-2}	7.39×10^{-5}	3.11×10^{-3}	1.05×10^{-3}
cis-Chlordane	90	8.52×10^{-4}	5.47×10^{-5}	1.76×10^{-3}	1.11×10^{-5}	1.54×10^{-5}	1.31×10^{-4}	9.38×10^{-4}	8.67×10^{-4}
trans-Chlordane	12	2.94×10^{-3}	2.76×10^{-3}	8.27×10^{-4}	5.01×10^{-4}	2.44×10^{-3}	7.59×10^{-4}	1.41×10^{-2}	6.49×10^{-3}
heptachlor	38.1	3.92×10^{-4}	1.15×10^{-4}	2.88×10^{-3}	1.29×10^{-4}	3.34×10^{-4}	3.35×10^{-4}	3.87×10^{-4}	9.71×10^{-5}
heptachlor epoxide isomer B	84	5.22×10^{-4}	2.46×10^{-4}	3.18×10^{-5}	6.08×10^{-5}	3.46×10^{-5}	1.33×10^{-4}	1.02×10^{-4}	7.75×10^{-4}
methoxychlor	150	1.62×10^{-4}	1.12×10^{-4}	1.17×10^{-4}	7.31×10^{-5}	6.78×10^{-4}	3.82×10^{-5}	1.16×10^{-4}	1.61×10^{-4}
mirex	1000	9.67×10^{-6}	8.55×10^{-7}	1.01×10^{-5}	1.24×10^{-6}	2.15×10^{-6}	2.30×10^{-6}	4.11×10^{-6}	5.68×10^{-6}
**ΣOCPs**		**1.03×10^{0}**	2.82×10^{-1}	**4.54×10^{0}**	1.16×10^{-1}	**1.49×10^{0}**	1.22×10^{-1}	**1.39×10^{0}**	**1.38×10^{0}**
